# A Case of Invasive Pulmonary Aspergillosis vs. Pulmonary Aspergilloma in Immunocompromised Patient With Preexisting Lung Cavity

**DOI:** 10.7759/cureus.28073

**Published:** 2022-08-16

**Authors:** John Pueringer, Philip Stephens, Nirzari K Pandya, Syed Zaidi

**Affiliations:** 1 Internal Medicine, Philadelphia College of Osteopathic Medicine (PCOM), Philadelphia, USA; 2 Internal Medicine, University of Pittsburgh Medical Center, Pittsburgh, USA

**Keywords:** chronic cavitary pulmonary aspergillosis, aspergilloma risk factors, cavitary lung lesion, pulmonary aspergillosis, invasive aspergillosis

## Abstract

Subacute invasive aspergillosis (SAIA) occurs in immunocompromised patients and/or patients with preexisting pulmonary pathology. An aspergilloma is a fungus ball that occurs in preexisting lung cavities and can be relatively asymptomatic without tissue invasion. In contrast to an aspergilloma, SAIA invades local tissue and parenchyma, resulting in tissue necrosis. We present a case of a 68-year-old immunocompromised female with a past medical history of hypertension, hyperlipidemia, chronic obstructive pulmonary disease (COPD), stage IIIA adenocarcinoma, and a preexisting pulmonary cavity with chronic invasive aspergillosis vs. pulmonary aspergilloma treated with oral (PO) voriconazole. This case demonstrates that invasive aspergillosis should be considered in the differential diagnosis of any pulmonary lung lesion showing tissue invasion and expansion in an immunocompromised patient.

## Introduction

Aspergillus is a ubiquitous airborne fungus that most people encounter daily, which may lead to various infectious and/or allergic diseases depending on the host's immune status or pulmonary structure. *Aspergillus fumigatus* (*A. fumigatus*) is the most prevalent human colonizer, though *A. niger*, *A. flavus*, and *A. oxyzae* are also reported to cause human diseases [1]. Pulmonary aspergillosis refers to several different conditions caused primarily by *A. fumigatus*.

Subacute invasive aspergillosis (SAIA) occurs in immunocompromised patients and/or patients with preexisting pulmonary pathology. Critically ill patients and those with COPD are particularly at increased risk [2]. Aspergilloma is a fungus ball that occurs in preexisting lung cavities and can be relatively asymptomatic without tissue invasion. In contrast to an aspergilloma, SAIA invades local tissue and parenchyma, resulting in tissue necrosis. 

This article was previously presented as a poster at PCOM's Research Day 2022 on May 11, 2022.

## Case presentation

A 68-year-old female presented to the ED in October 2021 with failure to thrive, productive cough, and was found to have a worsening of the right lower lobe (RLL) cavitary lesion in the setting of COPD and treated but not cured lung cancer. Past medical history was significant for stage IIIA adenocarcinoma of the right upper lobe (RUL) with regional lymph node, bone, and soft tissue metastasis. She underwent a neoadjuvant chemo and radiation therapy, followed by a right upper lobectomy with mediastinal dissection in 2014. She was also found to have a cavitary superior segment RLL lesion in 2019 following a bronchoscopy with biopsy, cytology, and cultures which only showed inflammatory changes without evidence of recurrence. She was treated empirically with antibiotics and then lost to follow-up during the pandemic. Significant comorbidities included hypertension, hyperlipidemia, and COPD. In September 2021, she was admitted for pneumonia with CT findings of a large right cavitary lesion, followed by a course of IV antibiotics and oxygen therapy. Social history included smoking a quarter of a pack of cigarettes daily for over 40 years with no significant alcohol usage. 

She presented to the ED in October 2021 with general malaise, failure to thrive, and chronic deconditioning. She endorsed poor appetite with anhedonia, a depressed affect, a productive cough, sacral wounds from immobility, and intermittent episodes of diarrhea and constipation along with her urinary incontinence that developed secondary to a UTI the month prior. No recent episodes of fevers, chills, chest pain, dyspnea, abdominal pain, nausea, or vomiting were reported.

ED vitals included a temperature of 98.1° F, pulse 92 beats/min, blood pressure 91/43 mmHg, oxygen saturation (SpO2) was 100% on 2 liters (L) nasal cannula, weight was 48 kg, and respiratory rate was 25 respirations/min. A physical exam revealed that she was cachectic, with temporal and muscle wasting. Her pulmonary exam was significant for unlabored breathing and coarse breath sounds greater in the right lung than the left. A cardiovascular exam revealed a regular rate and rhythm with S1 and S2. The abdomen was soft, non-tender, and non-distended with normal bowel sounds. The patient was able to move all extremities spontaneously, with no edema noted. She was alert with no focal neurological deficits but had a depressed affect and was slightly irritable. 

Laboratory studies were significant for hypocalcemia of ionized calcium of iCal 0.62 mg/dL, hypomagnesemia of Mg 0.4 mg/dL, leukocytosis with a neutrophil predominance of WBC 22,700/mm3, neutrophils 91.0%, elevated lactate at 4.6 mEq/L and elevated alkaline phosphatase at 413 U/L. Her remaining electrolytes and liver function enzymes were within normal limits. 

**Figure 1:**
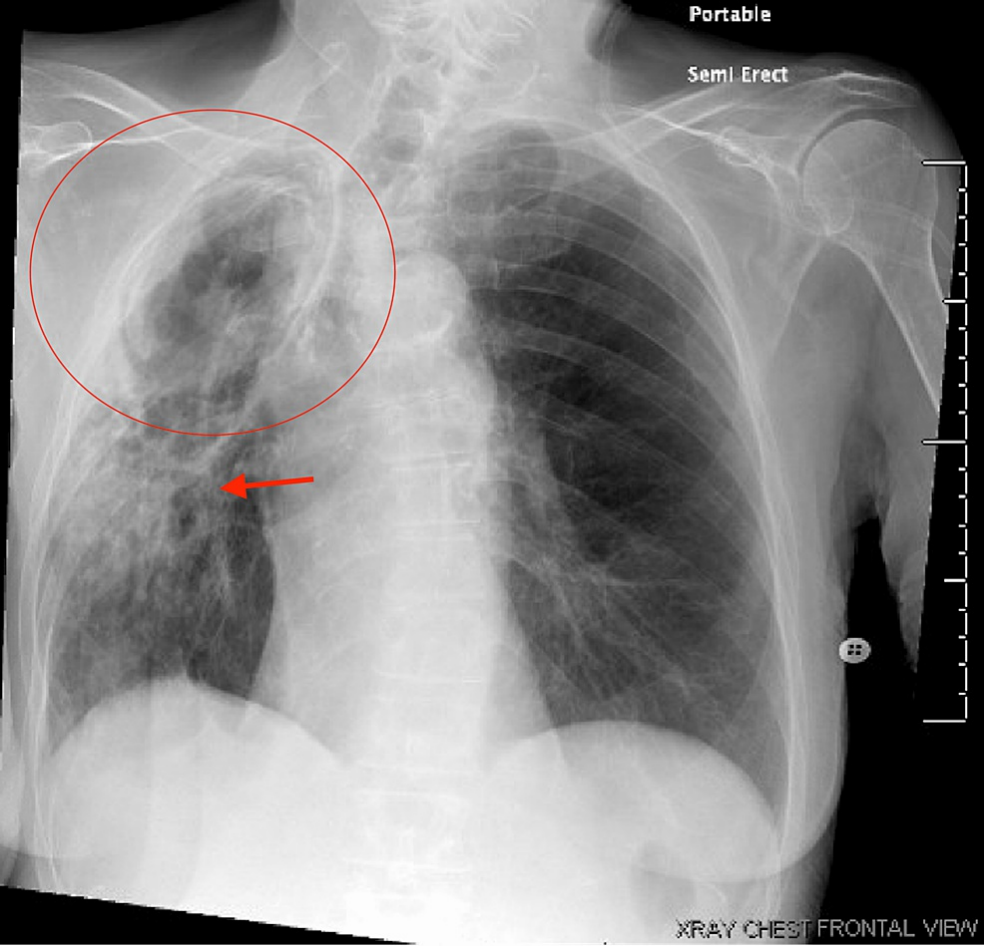
Chest radiograph showing cavitary lesion in the right upper lung lobe (circle) and new focal opacity (red arrow).

**Figure 2:**
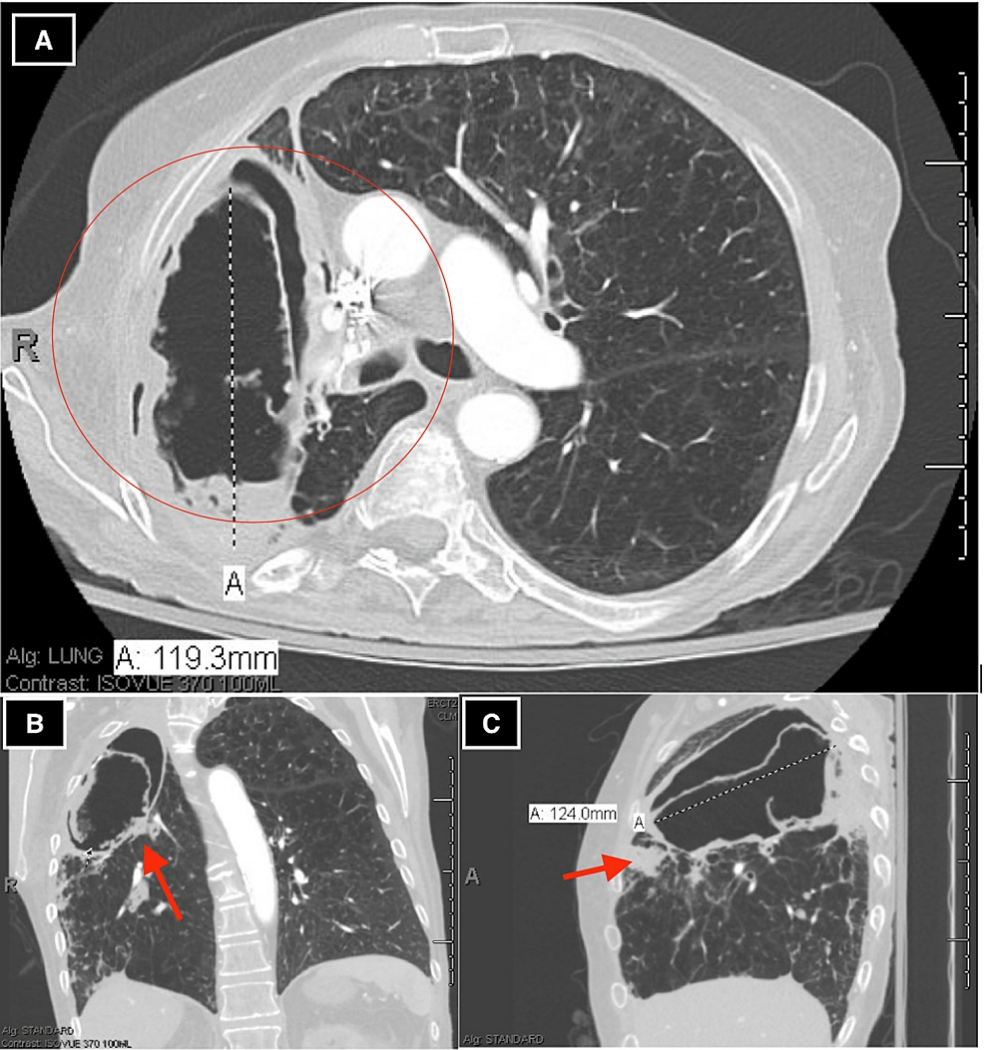
CT images of chronic invasive pulmonary aspergillosis. Thick-walled cavity in the right upper lung lobe (circle) with new internal round debris, thick rind, internal papillary projections, cauliflower-like lobulations, pericavitary consolidation and possible formation of a fungal ball within the cavity (red arrows) present in (A) axial view, (B) coronal view, and (C) sagittal view.

Chest radiograph revealed a new focal opacity in the right midlung with chronic cavitary changes in the right apex (Figure 1). CT chest revealed a 12 cm RUL cavity, mildly enlarged from 2019 with new internal round debris, which contained a thick rind and internal papillary projections, cauliflower-like lobulations, and chronic mild consolidative changes (Figure 2). 

In the ED, she received IV electrolyte repletion with 2 gm magnesium sulfate and 1 gm calcium gluconate along with 1.5 L IV fluids. In addition, she was started on broad-spectrum antibiotics with 750 mg IV vancomycin, which was renally dosed by pharmacy every 12 hours, 2 gm IV cefepime every 12 hours, and 500 mg PO metronidazole given three times daily. At this time, differential diagnoses included aspergilloma, SAIA, pulmonary anaerobic abscess, tuberculosis infection, recurrent aspiration in the superior segment, radiation-associated necrosis, and recurrence of malignancy. 

The patient was admitted to the medical ward and placed in isolation. Four subsequent sputum acid-fast bacillus (AFB) stains and mycobacterium cultures were negative, so antibiotics and isolation orders were discontinued after completing a 10-day course. Sputum cultures and a repeat of two samples were positive for *A. fumigatus*, mold, and occasional yeast. Serum Fungitell β-D-glucan, galactomannan and Aspergillus Ag (IgG) were all positive. Biopsy was considered; however, based on the patient's poor nutritional status as well as multiple comorbidities, it was thought to be safer to hold off on biopsy and treat empirically for invasive aspergillosis versus aspergilloma. Likewise, a right upper extremity deep vein thrombosis (DVT) was confirmed by ultrasound (US), and the patient was treated with apixaban. 

Treatment was started with voriconazole 200 mg taken orally with a dosing weight of 46.1 kg and anticipated to continue for at least 84 days. The patient was scheduled to repeat CT chest 4-6 weeks following discharge. Unfortunately, she was largely bedbound because of her oxygen requirement, severe malnutrition, and significant deconditioning. 

A month later, our patient was readmitted to the hospital with electrolyte derangements and found to have bilateral lower extremity DVTs that were deemed apixaban failure and subsequently started on enoxaparin. A follow-up CT chest in December 2021 showed no new acute abnormalities identified compared to the previous study in October 2021. However, it showed a stable-to-slight interval decrease in size of the area of cavitation in the superior segment of the RLL, now measuring 8.5 cm x 4.1 cm x 11.1 cm, which compared with previous measurements of 8.5 cm x 4.1 cm x 12 cm. Four months later, in March 2022, a further decrease in size of the cavitation reduced to 8.5 cm x 4.1 cm x 10.1 cm without any new acute abnormalities. Eventually, the patient died of cardiac arrest after admission to the hospital in May 2022 for a femur fracture.

## Discussion

SAIA occurs primarily in patients with some degree of immunocompromise with progressive features over 1-3 months [3]. Invasive disease is rare in immunocompetent patients as macrophages, neutrophils, and T cells rapidly neutralize and clear the organism. Patients with severe immunocompromise are far more likely to develop acute invasive disease [2]. It has become more significant in patients with malignancy, organ transplantation, autoimmune and inflammatory conditions. Critically ill patients and those with COPD are particularly at increased risk [2]. SAIA will also present with a cavity that may or may not contain an aspergilloma. Although the fungal mass in this form is similar to an aspergilloma, it represents a different process. Rather than Aspergillus colonizing a preexisting cavity, in SAIA, focally invasive aspergillosis occurs, which eventually undergoes central necrosis and cavitation, forming its own cavity. The finding of tissue invasion allows this entity to be distinguished from the more common aspergilloma [4]. Appearances may then be the same as an aspergilloma. 

Chronic pulmonary aspergillosis (CPA) is a destructive disease of the lung found in immunocompetent or mildly immunocompromised patients that can progress to tissue necrosis. The most common form of CPA is chronic cavitary pulmonary aspergillosis (CCPA), which, when left untreated, may progress to chronic fibrosing pulmonary aspergillosis [3]. Less common manifestations include an Aspergillus nodule and single aspergilloma. All these entities are found in immunocompetent patients with prior or current lung disease. The distinctive hallmark is new and expanding cavities with thick or thin walls associated with pulmonary and systemic symptoms, including signs of systemic inflammation over months to years [3]. An intracavitary fungal ball may be present, often with pleural thickening and extensive parenchymal destruction and/or fibrosis [5]. CPA is characterized by weight loss (>90%), cough, hemoptysis, and fatigue lasting for >3 months [6]. Hemoptysis is the most frequent symptom of aspergillosis due to direct invasion of capillaries of the wall lining, endotoxin release from the organism, and mechanical irritation of exposed vessels within the cavity [7, 8]. Increased expression of vascular endothelial growth factor (VEGF) leads to pulmonary arterial and bronchial dilation, possibly subjecting them to erosion and thus hemoptysis [9].

A pulmonary aspergilloma is a form of pulmonary involvement of Aspergillus, defined as an infection without invasion of preexisting lung cavities [10]. Aspergillus spores are inhaled most commonly through the respiratory tract and thrive in poorly drained and avascular cavitary spaces [1]. Once harbored within cavitary airspace, it adheres to the wall with its conidia, then converts to potentially pathogenic hyphae and evokes an inflammatory reaction mixed with mucous and cellular debris [1]. Aspergilloma is more common in immunocompetent patients with preexisting lung disease, while immunocompromised patients are more often inclined to develop invasive Aspergillus infections [1]. The fungal mass may move within the cavity on radiologic imaging (called the Monod sign) [11]. Aspergillus does not usually invade the surrounding lung parenchyma or vasculature in this setting. Most patients are asymptomatic but may develop hemoptysis or other exceptions [3, 12]. The lesion is stable in most cases but can decrease in size or resolve spontaneously without treatment in 10% of cases [12]. Rarely does the aspergilloma increase in size, but risk factors for poor prognosis include the severity of lung disease, size of the cavity, and immunosuppression. 

According to the 2020 European Organization for Research on Treatment of Cancer and the Mycoses Study Group Education and Research Consortium (EORTC-MSGERC) criteria, probable invasive fungal diseases (IFDs) require the presence of at least one host factor (i.e., neutropenia, hematologic malignancy, solid organ transplant, etc.), a clinical feature (i.e., features of pulmonary aspergillosis, tracheobronchitis, central nervous system infection, etc.), and mycologic evidence (i.e., any mold recovered on culture by sputum, bronchoalveolar lavage (BAL), aspirate, or presence of galactomannan, etc.), and is proposed for immunocompromised patients, as evidenced in Table 1 [13]. Recent advances in diagnostic testing include serum Aspergillus antigenic cell wall biomarkers, galactomannan, β-D-glucan, and IgG antibodies against Aspergillus, all of which were positive in our patient. Galactomannan is a polysaccharide released by Aspergillus during growth [2]. A meta-analysis showed that the galactomannan assay had a sensitivity of 71% and specificity of 89% for invasive pulmonary aspergillosis, with a negative predictive value of 92-98% but a positive predictive value of only 25-62% [14]. Galactomannan antigen was incorporated in the revised EORTC-MSGERC criteria for diagnosing invasive pulmonary aspergillosis. As part of the mycotic evidence for invasive aspergillosis, galactomannan antigen needs to be collected in plasma, serum, BAL, or CSF. Specifically, obtaining a single serum or plasma ≥1.0, BAL fluid ≥1.0, single serum or plasma ≥0.7 and BAL fluid ≥0.8, and/or CSF ≥1.0 [13], β-D-glucan was also shown to have similar sensitivity, specificity, and positive and negative predictive values for this test in diagnosing invasive disease [15]. In addition, IgG antibodies against Aspergillus species diagnosed by precipitin assay are positive in over 90% of chronic cases [3, 5].

**Table 1:** Probable invasive pulmonary mold diseases. BAL: Bronchoalveolar lavage; CSF; Cerebrospinal fluid; PCR: Polymerase chain reaction. *Hematologic malignancy refers to active malignancy, in receipt of treatment for this malignancy, and those in remission in the recent past. These patients would comprise largely acute leukemias and lymphomas, as well as multiple myeloma, whereas patients with aplastic anemia represent a more heterogeneous group of individuals and are not included.

Host factors
Recent history of neutropenia (<0.5 × 10^9^ neutrophils/L [<500 neutrophils/mm3] for >10 days) temporally related to the onset of invasive fungal disease
Hematologic malignancy*
Receipt of an allogeneic stem cell transplant
Receipt of a solid organ transplant
Prolonged use of corticosteroids (excluding among patients with allergic bronchopulmonary aspergillosis) at a therapeutic dose of ≥0.3 mg/kg corticosteroids for ≥3 weeks in the past 60 days
Treatment with other recognized T-cell immunosuppressants, such as calcineurin inhibitors, tumor necrosis factor-a blockers, lymphocyte-specific monoclonal antibodies, immunosuppressive nucleoside analogues during the past 90 days
Treatment with recognized B-cell immunosuppressants, such as Bruton’s tyrosine kinase inhibitors, eg, ibrutinib
Inherited severe immunodeficiency (such as chronic granulomatous disease, STAT 3 deficiency, or severe combined immunodeficiency)
Acute graft-versus-host disease grade III or IV involving the gut, lungs, or liver that is refractory to first-line treatment with steroids
Clinical features
*Pulmonary aspergillosis*
The presence of one of the following four patterns on CT:
Dense, well-circumscribed lesions(s) with or without a halo sign
Air crescent sign
Cavity
Wedge-shaped and segmental or lobar consolidation
*Other pulmonary mold diseases*
As for pulmonary aspergillosis but also including a reverse halo sign
*Tracheobronchitis*
Tracheobronchial ulceration, nodule, pseudomembrane, plaque, or eschar seen on bronchoscopic analysis
*Sino-nasal diseases*
Acute localized pain (including pain radiating to the eye)
Nasal ulcer with black eschar
Extension from the paranasal sinus across bony barriers, including into the orbit
*Central nervous system infection*
One of the following two signs:
Focal lesions on imaging
Meningeal enhancement on MRI or CT
Mycological evidence
Any mold, for example, *Aspergillus*, *Fusarium*, *Scedosporium* species or Mucorales recovered by culture from sputum, BAL, bronchial brush, or aspirate
Microscopical detection of fungal elements in sputum, BAL, bronchial brush, or aspirate indicating a mold
*Tracheobronchitis*
Aspergillus recovered by culture of BAL or bronchial brush
Microscopic detection of fungal elements in BAL or bronchial brush indicating a mold
*Sino-nasal diseases*
Mold recovered by culture of sinus aspirate samples
Microscopic detection of fungal elements in sinus aspirate samples indicating a mold
*Aspergillosis only*
*Galactomannan antigen*
Antigen detected in plasma, serum, BAL, or CSF
Any 1 of the following:
Single serum or plasma: ≥1.0
BAL fluid: ≥1.0
Single serum or plasma: ≥0.7 and BAL fluid ≥0.8
CSF: ≥1.0
*Aspergillus PCR*
Any 1 of the following:
Plasma, serum, or whole blood 2 or more consecutive PCR tests positive
BAL fluid 2 or more duplicate PCR tests positive
At least 1 PCR test positive in plasma, serum, or whole blood and 1 PCR test positive in BAL fluid
*Aspergillus* species recovered by culture from sputum, BAL, bronchial brush, or aspirate​​​​​​​

The specific criteria used for the diagnosis of CPA are a large cavity or more on chest imaging with or without a fungal ball (aspergilloma) in one or more of the cavities and at least one of the following symptoms for at least three months: fever, weight loss, fatigue, cough, sputum production, hemoptysis, or shortness of breath - additionally, a positive Aspergillus IgG with or without a culture of Aspergillus from the lungs [3]. If a fungal ball is observed, then confirmation that Aspergillus is responsible requires only an Aspergillus IgG or precipitins test to be positive, which is usually the case [3]. When a single aspergilloma is suspected, if the aspergilloma is single, the cavity stable over months, and the patient has few symptoms and little evidence of systemic inflammation, a simple aspergilloma may be diagnosed.

In this case study, there were overlapping features of aspergilloma and SAIA. Our patient presented with some important risk factors in classifying this disease. She had a preexisting cavity, given her history of metastatic adenocarcinoma of the lung, radiation therapy, and subsequent RUL lobectomy. Therefore, it is possible her cavity appeared to expand due to infection, malignancy, and/or recurrent aspiration. To this point, diagnostic workup failed to demonstrate other infections and/or recurrent cancer. The significance of isolating Aspergillus in sputum samples depends on the patient's immune status. In immunocompetent patients, it almost always represents colonization with no clinical consequences. However, isolation of Aspergillus species in sputum culture in immunocompromised patients is highly suggestive of invasive species. In patients with hematologic malignancy, granulocytopenia, hematopoietic stem-cell transplant, or a bone-marrow transplant, positive predictive cultures can range from 70 to 90% [2, 16].

We could isolate mold and moderate Aspergillus in culture, specifically *A. fumigatus*, which was found to be susceptible to voriconazole on susceptibility testing. Based on the 2020 EORTC-MSGERC criteria, our patient has the presence of malignancy, clinical features of Aspergillosis on CT, including a new cavity, and mycologic evidence, including galactomannan antigen and Aspergillus, collected on sputum culture, making this case a probable invasive fungal disease. Traditionally, bronchoscopy with biopsy demonstrating tissue invasion is considered the gold standard for diagnosis. In this case, a biopsy was considered; however, based on the patient's positive culture results, poor nutritional status, and multiple comorbidities, it was thought to be safer to hold off on biopsy and treat empirically for invasive Aspergillus.

It is essential to classify the disease to help decide the therapy. For example, in a patient with Aspergilloma, the treatment approach has leaned towards surgical resection for Aspergilloma, if possible, or arterial embolization in cases of severe hemoptysis. Alternatively, antifungal therapy, such as itraconazole 200-400 mg, provides some benefit for treating a simple aspergilloma [17]. Many patients, such as those who are asymptomatic and have stable radiographic findings over many months, require no therapy. However, with invasive forms of CPA, the current treatment of choice is triazole medication, such as voriconazole 200 mg or posaconazole 300 mg [18]. Voriconazole is indicated for at least 6-12 weeks until lesions and immunosuppression resolve [3, 5, 19]. Voriconazole has a milder side-effect profile and is much better tolerated than the older first-line treatment, amphotericin B. However, voriconazole is an inhibitor of the cytochrome P450 (CYP 450) enzyme. It thus is associated with a significant number of drug-drug interactions, such as with cyclosporine, warfarin, terfenadine, carbamazepine, quinidine, rifampin, statins, direct oral anticoagulants, and sulfonylureas [20, 21]. An important drug interaction to highlight in this case is voriconazole and apixaban. Apixaban is an anticoagulant whose mechanism is a direct factor Xa inhibitor, which decreases clot growth and thrombus formation and is used for the treatment of DVT. Apixaban is metabolized primarily by the cytochrome P450 (CYP 450) system and subsequently renally excreted [22]. This is especially important in our patient, who was started on and discharged with, apixaban, secondary to an upper extremity DVT that she developed during her hospitalization. The readmission of our patient with bilateral lower extremity DVTs was deemed an apixaban failure. However, the likely explanation for apixaban failure is decreased metabolism from CYP 450 system due to inhibition from voriconazole. Therefore, in patients started on voriconazole, who are at increased risk for DVTs or need treatment for DVT, it would be strongly suggested to find alternatives to anticoagulation medications that are metabolized by the CYP 450 system (e.g., warfarin and apixaban) or monitor anti-factor Xa levels. In addition, continued observation after initial therapy for Aspergillus is recommended. The prognostic method of choice is a low-dose CT chest every 6-12 months in immunocompetent patients. It is critical to follow the IgG titer. A rising Aspergillus IgG titer is indicative of treatment failure [1]. These are all especially important to this case because our patient has a large cavity secondary to a right upper lobectomy and immunocompromised state. Therefore, it is unclear whether or not there is enough vasculature for the antifungal medication to reach the fungal infection and provide treatment. In our case, we could not obtain a follow-up IgG titer value. However, follow-up CT imaging at eight weeks revealed decreased cavity size on PO voriconazole therapy and improved symptoms, including increased strength, occasional cough, and no hemoptysis or sputum production.

## Conclusions

Aspergillus syndromes may coexist or may progress from one entity to the other. This case presented a patient with a large preexisting pulmonary cavity, immunocompromised state, and enlarging opacity on imaging, suggesting a SAIA vs. Aspergilloma. The invasion into the surrounding parenchyma and enlarging cavity provides more evidence of the former. In addition, sputum cultures and serum Aspergillus antigenic cell wall biomarkers - galactomannan, β-D-glucan, IgG antibodies - were all positive for Aspergillosis, which is highly suggestive of invasive disease in an immunocompromised state. Treatment was initiated with 12 weeks of PO voriconazole, follow-up CT chest every 6-12 months, and follow-up IgG titers. In addition, the use of enoxaparin for the treatment of DVT when a patient is on voriconazole is preferred to reduce drug-drug interactions and avoid toxicity or failure with other CYP 450 metabolized anticoagulants (e.g., apixaban and warfarin). 
